# Supplementary material for: A latent profile analysis of drinking motives among graduate student heavy drinkers
Source: Addict Behav Rep. 2025 Sep 13;22:100630. doi: 10.1016/j.abrep.2025.100630 (PMC12481705; doi:10.1016/j.abrep.2025.100630)
Supplement: Supplementary Data 1 [file mmc1.docx]

**Abstract**

Person-centered analyses have been used to identify subgroups of drinkers based on their drinking motivations and associations with problematic drinking behaviors. Graduate students engage in heavy drinking patterns; however, no research has examined these associations between drinking motives and drinking patterns in graduate students. The current study aimed to: a) identify profiles of graduate student drinkers by endorsement of drinking motives, and b) explore associations between profiles and alcohol use and alcohol-related consequences. Participants consisted of 325 graduate students, primarily white (71.3%), 54.9% female, with a mean age of 26 years old. A latent profile analysis revealed five distinct profiles: high levels of all motives; low levels of all motives; moderate levels of all motives; high levels of external motives; and a mixed level of motives. Negative binomial regression analyses revealed significant differences between profile membership, and both drinks per week and alcohol-related consequences. These findings may be used to inform interventions to reduce problematic drinking behaviors among graduate students by encouraging practitioners to target those with profile memberships that are associated with higher drinking levels.

Keywords: graduate students, drinking motives, drinking

**A Latent Profile Analysis of Drinking Motives Among Graduate Student Drinkers**

Many individuals “mature out” of heavy drinking after graduating college with an undergraduate degree, as their adult responsibilities increase (Jochman & Fromme, 2010). However, graduate students continue their education during emerging adulthood, typically spanning ages 18 to 29. Through this developmental period, graduate students are in a unique position where they are faced with adult responsibilities, identity exploration, and transitioning roles, while still being in a college environment (Arnett, 2000). During this time, many graduate students engage in regular drinking: specifically, 58% of graduate students reported drinking alcohol in the past 30 days (ACHA, 2019). In addition, approximately 11.3% of graduate students meet criteria for heavy drinking (ACHA, 2019). On the other hand, approximately 7.9% of the same aged general adult population meets criteria for heavy drinking (SAMHSA, n.d.). Thus, graduate students may not “mature out” of heavy drinking after undergraduate education: instead, they may continue to engage in these problematic patterns.

One potential reason for graduate students engaging in heavy drinking is to cope with ongoing stressors (El-Ghoroury et al., 2012; Offstein et al., 2004). Emerging adulthood is a time where many report heightened levels of stress due to financial concerns, academic pressures, and making independent decisions (Arnett, 2007). For many emerging adults, the COVID-19 pandemic further taxed their mental health with the addition of stressors such as social isolation/distancing and institutional closures, hindering their ability to increase their personal responsibilities and make independent decisions (Arnett & Mitra, 2024). Unsurprisingly, 43% of emerging adults screened positive for anxiety disorders and 38% screened positive for major depression during the pandemic (Arnett & Mitra, 2024). This increased risk for mental health symptoms may be due to the disruption in developmental features such as self-exploration, instability, and self-focus during COVID-19 (Arnett & Mitra, 2024). Thus, it is not surprising that 43% of graduate students report coping with their stressors by engaging in alcohol consumption (Ickes et al., 2015). However, drinking to cope increases the risk of developing an alcohol use disorder in the future (Littlefield et al., 2010). Thus, it is important to further understand risk factors for heavy alcohol use to inform future prevention and intervention efforts for graduate students.

Examining drinking motives has aided researchers in better understanding drinking behaviors and identifying risk factors for alcohol use disorders in the future (Cooper, 1994). Specifically, there are four types of drinking motives that are commonly conceptualized through their valence (positive/approach and negative/avoidance) and source (internal/self or external/other) of one’s expectations of drinking (Cox & Klinger, 1988). Many individuals drink to enhance positive affect or reduce negative affect (i.e., valence), and drink in response to internal rewards (e.g., emotions), or external rewards (e.g., social acceptance; Cox & Klinger, 1988). The four drinking motives based on their valence and source can be described as the following: enhancement motives (i.e., internal, positively reinforcing), coping motives (i.e., internal, negatively reinforcing), social motives (i.e., external, positively reinforcing), and conformity motives (i.e., external, negatively reinforcing; Cooper, 1994).

Drinking motives have unique associations with drinking outcomes, such that internally focused motives are associated with higher risk outcomes when compared to externally focused motives. For instance, individuals who drink to cope have the highest level of alcohol-related consequences, when compared to other motives (Cooper, 2015). Individuals who drink for enhancement reasons report higher alcohol-related consequences, which is mediated by higher levels of alcohol consumption (Cooper et al., 2015). Social motivation shows moderate associations with alcohol consumption and alcohol-related consequences, suggesting non-problematic drinking behaviors (Cooper, 2015). Meanwhile, high endorsement of conformity motivation has weak negative associations with alcohol use, and weak positive associations with alcohol-related consequences (Cooper, 2015; Grant et al., 2009).

Most of the research examining the association between drinking motives and drinking patterns has focused on variable-level analyses. Person-centered analyses are better at determining specific subgroups of individuals who share similar traits (Coffman et al., 2007). Identifying subgroups of drinkers who are at a higher risk for higher alcohol consumption and alcohol-related consequences can inform which type of drinkers interventions should target. A common person-centered approach is latent profile analysis (LPA), which allows researchers to identify subgroups (i.e., profiles) in their sample based on similar characteristics, which can then be used to predict various outcomes. Identifying subgroups and determining how they differ in terms of problematic drinking behaviors can provide a more nuanced understanding of the complex interplay between drinking motives and problematic drinking behaviors that may be missed when using a variable centered approach. This is especially important as drinking motives do not occur independently, as many will drink for multiple motivations (Cooper, 1994). Exploring subgroups of graduate students via drinking motives can help researchers develop more tailored interventions for this population.

There are several distinct profiles that exist among emerging adults based on their drinking motivations (Cadigan et al., 2015; Coffman et al., 2007; Holt et al., 2013). Multi-reasoners (i.e., extreme endorsement of all motives) have a higher frequency of drunkenness and more alcohol-related consequences in comparison to other profiles (Coffman et al., 2007; Holt et al., 2013). Similarly, those who endorse average levels of all drinking motives, and have average levels of drinking patterns, report the highest alcohol-related consequences (Holt et al., 2013). Although drinkers with this profile do not engage in heavy alcohol consumption, they may be more at risk for heavy drinking in the future because they experience the highest levels of alcohol-related consequences and have unique vulnerability dimensions (i.e., high negative affect, low social support; Holt et al., 2013). In addition, profiles are often examined to the extent that the motives endorsed are positively or negatively reinforcing. Individuals who highly endorse positively reinforcing motives (i.e., enhancement and social) report the highest levels of alcohol consumption (Cadigan et al., 2015). Uniquely, those who endorse high levels of positively reinforcing motives and coping motives, report experiencing the highest levels of alcohol-related consequences (Cadigan et al., 2015). Taken together, individuals who highly endorse more than one drinking motivation show higher levels of alcohol consumption, and alcohol-related consequences (Cadigan et al., 2015).

There is limited data demonstrating the association between drinking motives and outcomes in populations beyond undergraduate students at the variable or person level. The existing research with graduate students suggests a tendency to endorse social and enhancement motivation for drinking most often, followed by conformity and coping motivation (Allen et al., 2020). Graduate students who drink for enhancement and social reasons report higher levels of alcohol quantities, and those who drink for conformity reasons report lower levels of alcohol use frequency (Allen et al., 2020). Uniquely, those who drink for coping reasons report higher levels of alcohol quantity and higher alcohol use frequency (Allen et al., 2020). In addition, adults (i.e., ages 18-65) most often endorse enhancement and social motivations who in turn report moderate alcohol use; meanwhile, adults who endorse coping motivation report hazardous alcohol use (D’Aquino et al., 2023; Mezquita et al., 2011). Unfortunately, there is a lack of research examining the associations between drinking motives and alcohol-related consequences for graduate students, or the association between these factors at a person-centered level.

**Current Study**

Graduate students engage in problematic drinking behaviors. Understanding predictors of higher levels of alcohol use and alcohol-related consequences can help inform intervention efforts for this population. Drinking motives have been extensively examined in adolescents and emerging adults, particularly the influence of drinking motives on drinking patterns at the variable level. Researchers using person-centered approaches have found that subgroups of drinkers who highly endorse various motives report the highest levels of alcohol use and alcohol-related consequences. Using a person-centered approach allows for the identification of naturally occurring subgroups within a sample of graduate students and may reveal nuanced patterns that may be overlooked when using a variable-centered analyses. However, no research has examined the association between graduate students’ drinking motives and drinking patterns at the person level. The current study aimed to 1) identify profiles of graduate student drinkers based on their endorsements of drinking motives, and 2) explore the associations between different profiles and alcohol use and alcohol-related consequences. Based on past evidence, a potential profile will include graduate students who drink for social and enhancement reasons and may report moderate levels of alcohol use (Allen et al., 2020; D’Aquino et al., 2023; Mezquita et al., 2011).

**Methods**

Data collection for this study occurred throughout the fall of 2021, with participants recruited from various social media platforms (e.g., Facebook, Reddit; 87%) and a daily email announcement through a university listserv (13%). All study materials and procedures were approved by the Institutional Review Board of the first author. Inclusion criteria to participate included the following: at least 18 years old and enrolled in a current graduate program in the United States. In particular, participants were asked if they were currently enrolled in a graduate program in the United States, and if a participant answered “no” they were not included in the study. Recruited participants went through informed consent procedures and completed a CAPTCHA prompt to ensure study security. Next, a baseline assessment was provided to participants to gather information on their demographics, drinking patterns, and various health behaviors. Participants received compensation in the form of $20 Amazon e-gift cards if they correctly answered two out of three check questions (i.e., Please select disagree for this question).

**Participants**

The final sample consisted of 331 participants who were concurrently enrolled in a graduate program at a northeastern university in the United States and had a mean age of 26.2 years old (*SD*= 3.3) ranging from 18 to 41 years old. Most participants identified as female (54.9%) and non-Hispanic white (52.6%). Participants also identified as Hispanic white (19%), Black (20.8%), Native American or Native Alaskan (5%), Asian (2%), Native Hawaiian or Pacific Islander (0.3%), and other (0.3%). Participants also reported the fields in which they are earning their graduate degree, with psychology (18.6%) being the most prevalent, followed by nursing (12.2%), business (9.4%), and computer science (8.5%). Participants reported a range of 1 to 6 years regarding time for program completion with a mean of 3.2 (*SD* = 1.2) years, and on average participants were 1.4 (*SD* = 1.4) years into their program.

**Measures**

***Drinks per week***

Participants completed the Daily Drinking Questionnaire (DDQ; Collins et al., 1985) to assess their drinking behaviors. This measure includes items that ask participants the quantity of alcohol they drink weekly (i.e., how many drinks per week), the frequency of alcohol consumption (i.e., how many days per week), and how many drinks they consume during each drinking event (i.e., how many drinks per typical occasion). The definition of one drink was outlined as follows and presented on a visual graphic: “For all questions, one drink equals: 5 oz. wine, 12 oz. wine cooler, 12 oz. beer (10oz. of Microbrew; 8-9 oz. Malt Liquor, Canadian beer or Ice beer), 1 Cocktail with 1 oz. of 100 proof liquor or 1 ½ oz. (single jigger) of 80 proof liquor.”.

***Alcohol-Related Consequences***

Alcohol-related consequences were assessed using the 24-item version of the Brief Young Adult Alcohol Consequences Questionnaire (BYAACQ; Read et al., 2006). This measure is tailored for use with college students and evaluates the previous month of drinking. Participants responded to each item on a dichotomous scale of yes (1) or no (0). A composite score was computed by adding how many items participants responded “yes.” Example items include: “While drinking, I have said or done embarrassing things”, “I have passed out from drinking”, and “I have often found it difficult to limit how much I drink” (Cronbach’s α = .89).

***Drinking Motives***

The Drinking Motives Questionnaire (DMQ; Cooper, 1994) was used to assess participants’ motivations for drinking. This measure includes 20 items on a 5-point Likert scale from 1 (Never/Almost Never) to 5 (Almost Always/Always). Items are intended to assess motivations for drinking in four areas such as: 1) enhancement (example items: “because you like the feeling”, “to get high”; α = .71), 2) social (example items: “because it helps you enjoy a party”, to be sociable”; α = .79), 3) conformity (example items: “because your friends pressure you to drink”, “to fit in with a group you like”; α = .68), and 4) coping (example items: “to forget your worries”, “to cheer up when you are in a bad mood”; Cronbach’s α = .71).

**Statistical Analysis Plan**

All statistical analyses were conducted in R Studio. First, descriptive statistics and Pearson correlations between all variables (i.e., drinking motives, drinks per week and alcohol-related consequences) were examined. Sixteen participants who reported zero drinks per week were excluded from the analyses. Next, a latent profile analysis (LPA) was used to determine the ideal number of profiles, which included the four drinking motives. An LPA determined the best fitting number of latent subgroups based on specific variables within the data. A 2-7 latent profile solution was run to determine the best fitting model. Determining the best fitting model was based on examining various model fit indices. In particular, we examined the Akaike information criterion (AIC), Bayesian information criterion (BIC), the Lo-Mendell-Rubin adjusted likelihood ratio test (LRT), and entropy, all of which offer insight into the model fit (Collins & Lanza, 2009; Lanza et al., 2012). AIC and BIC scores indicated a better fitting model. Entropy is deemed satisfactory when it is above .80. The LRT statistic compares the current model with a *k* -1 alternative, with a non-significant p-value (p > 0.05) suggesting acceptance of the previous model. LPA does not have a cut-off on class sizes; however, it was determined that classes with less than 5% membership would be difficult to interpret (Russel et al., 2022). Only classes with sufficient class membership (i.e., membership required more than 5% of the participants) was selected for interpretation. Additionally, we considered theoretical expectations and profile interpretability in determining the optimal number of profiles.

Next, likelihood ratio tests (LRT) were first conducted to assess the omnibus association between profile membership and each outcome, by comparing a reduced model (excluding profile membership) to a full model (including profile membership). Profile membership was saved from the LPA and used as the independent variable in these models. The reduced and full models used negative binomial regressions models because both outcome variables were count variables and positively skewed. When the LRT was significant, Tukey-adjusted post-hoc comparisons were conducted based on model-derived estimates from the negative binomial regressions. The post-hoc tests provided pairwise comparisons among all the profiles, while accounting for the use of count data and controlling for the family-wise error rate. Two post hoc power analyses were conducted to assess the adequacy of the sample size for detecting a significant effect in the negative binomial regression models. Using the Wald test, the estimated power was 0.99 for both models, where the beta levels for each negative binomial regression were used as the measure of effect size.

**Results**

**Determination of Number of Profiles**

Descriptive data and intercorrelations between motives, drinks, and consequences are presented in Table 1. We compared models containing between two and seven profile solutions based on fit indices (Table 2). Based on various model fit indices, the six-profile solution was the best fit (see Table 1 in supplemental materials). However, one profile had a class size below 5% membership, resulting in the use of the next best fitting model, the five-profile solution. The mean class posterior probabilities for the 5-profile solution comprised: class 1 as .97, class 2 as .91, class 3 as .94, class 4 as .86, and class 5 as .93.

**Latent Profile Characteristics**

The final model chosen was the five-profile solution, which revealed five distinct and interpretable classes. As seen in Figure 1 and in Table 3, there were distinguishable indicator response patterns for all five of the latent profiles. ***Moderate Endorsers*** (31% of sample) represented a group of students who reported moderate endorsement of all drinking motives. Specifically, graduate students in this profile had mean scores for each drinking motive ranging from 2.2 (some of the time) to 2.8 (half the time). ***Low Endorsers*** (9% of sample) represented a group of students who reported low endorsement of all drinking motives. Together, participants in this profile had mean scores for each drinking motives ranging from 1.2 to 1.6 (almost never). ***High Endorsers*** (30% of sample) represented a group of students who reported high endorsement of all drinking motives. Together, participants in this profile had mean scores for each drinking motives ranging from 3.2 to 3.4 (half the time). ***Conformity Endorsers*** (24% of sample) represented a group of students who reported a mix in endorsement of all drinking motives, with conformity motives endorsed the most. Specifically, students in this profile were likely to endorse coping, social and enhancement motives some of the time, and conformity motives half the time. ***External Endorsers*** (6% of sample) represented a group of students who reported a high endorsement of external drinking motives (i.e., social and conformity). Specifically, students in this profile were likely to endorse coping and enhancement motives some of the time, and social and conformity motives half of half of the time to most of the time.

**Profile Membership’s Association with Alcohol Use and Related Consequences**

After identifying five distinct profiles, we examined the degree to which different profiles are associated with alcohol use and alcohol-related consequences. First, LRT was conducted to examine how profile membership was associated with drinks per week. The negative binomial regression model including profile membership (full model) demonstrated better fit compared to the null model χ²(1) = 14.84, *p* < .001. Next, Tukey-adjusted post-hoc comparisons were conducted based on model-derived estimates from the full model. Tukey-adjusted post-hoc comparisons can be found in Table 4. There were significant differences in drinks per week between Low Endorsers, and all other profiles, such that Low Endorsers reported significantly lower drinks per week compared to Moderate Endorsers (*p* < .001), High Endorsers (*p* < .001), Conformity Endorsers (*p* < .001), and External Endorsers (*p* < .001). In addition, significant differences between Moderate Endorsers profile, Low Endorsers, High Endorsers, and External Endorsers were found, such that Moderate Endorsers had significantly higher reported drinks per week compared to Low Endorsers (*p* < .001) and significantly lower reported drinks per week compared to High Endorsers (*p* <.01) and External Endorsers (*p* < .05).

Next, a LRT was conducted to examine how profile membership was associated with alcohol-related consequences. The negative binomial regression model including profile membership (full model) demonstrated better fit compared to the null model χ²(1) = 96.10, *p* < .001. Next, Tukey-adjusted post-hoc comparisons were conducted based on model-derived estimates from the full model. Tukey-adjusted post-hoc comparisons can be found in Table 4. . There were significant differences between those in the Low Endorsers profile and all other profiles, such that Low Endorsers reported significantly fewer alcohol-related consequences compared to Moderate Endorsers (*p* < .001), High Endorsers (*p* < .001), Conformity Endorsers (*p* < .001), and External Endorsers (*p* < .001). In addition, significant differences between Moderate Endorsers, and Low Endorsers and High Endorsers, such that Moderate Endorsers reported significantly more alcohol-related consequences compared to Low Endorsers (*p* < .001), and significantly less alcohol-related consequences compared to High Endorsers (*p* <.001).Lastly, significant differences were found between High Endorsers and External Endorsers and Conformity Endorsers, such that High Endorsers reported significantly more alcohol-related consequences compared to External Endorsers (*p* < .01), and High Endorsers reported significantly more alcohol-related consequences compared to Conformity Endorsers (*p* = .05).

**Discussion**

The current study aimed to identify different profiles of graduate student drinkers by their endorsement of drinking motives and how those profiles were associated with problematic drinking behaviors. Five distinct profiles were identified including those who endorsed 1) moderate levels of all motives, 2) low levels of all motives, 3) high levels of all motives, 4) a mixed level of motives, and 5) high levels of external motives. In particular, the results for High Endorsers, which included graduate students who highly endorsed all drinking motives, are consistent with past findings that drinkers tend to drink for multiple reasons (Coffman et al., 2007; Holt et al., 2013). On the other hand, there were no profiles that clustered around negative reinforcement, which is inconsistent with past research (Cadigan et al., 2015). These results are consistent with findings that negatively reinforcing motives are less endorsed by graduate students compared to positively reinforcing motives (Allen et al., 2024). Of note, one profile (i.e., External Endorsers) showed high endorsement of external motives (i.e., social and conformity motives), suggesting a cluster of graduate students drink primarily in response to external rewards.

The five unique profiles demonstrated significant differences in drinking patterns (i.e., drinks per week, and alcohol-related consequences). Specifically, External Endorsers reported the highest drinks per week; however, did not have the highest report of alcohol-related consequences and had lower endorsement of coping motivation when compared to other profiles. This suggests External Endorsers engage in less risky drinking compared to other groups (i.e., High Endorsers) due to lower levels of alcohol-related consequences and coping motivation. Participants in the Low Endorsers profile reported the lowest drinks per week. In a similar manner, High Endorsers reported the highest number of alcohol-related consequences, and participants in the Low Endorsers profile reported the lowest alcohol-related consequences. Our findings are consistent, in that graduate students who have a low endorsement of all motives are the least at risk for problematic patterns of drinking (Cooper, 2015). In addition, High Endorsers reported the highest alcohol-related consequences which is consistent with the notion that multiusers report the highest level of alcohol-related consequences (Coffman et al., 2007; Holt et al., 2013). High Endorsers were also the profile who had the highest endorsement of coping motivation, which is consistently linked to greater alcohol-related harm (Cooper, 2015).

Similarly, our finding that graduate students who highly endorse external rewards drank the most drinks per week is consistent with the notion that graduate students who drink for social reasons report higher levels of alcohol consumption (Allen et al., 2020). On the other hand, the endorsement of external motives tends to be less risky than high endorsement of internal motives which is inconsistent with our findings that graduate students who highly endorse external motives are more likely to engage in risky drinking patterns (Cooper, 2015). One explanation for these findings is that endorsing high levels of external motives leads to risky drinking patterns, because of the social environment of graduate education. In graduate school, students tend to be in smaller cohorts thus having more pressure to drink in social situations (i.e., networking), resulting in many graduate students drinking for social or conformity reasons. In a similar manner, many students have a poor sense of belonging in the beginning which may lead students to conform with those around them to fit it, explaining why this profile (i.e., five) shows higher levels of alcohol consumption (Lambert et al., 2013). While this group represented a small proportion of our sample (i.e., 6%), its distinct motivational pattern and theoretical coherence suggest it may reflect a qualitatively distinct subgroup. However, we acknowledge that this finding should be interpreted cautiously and encourage future research to replicate this pattern in larger samples.

Although the implications of the current study are novel, there are limitations that should be noted. First, the current study used a cross-sectional design, limiting the ability to identify any causal changes between drinking motives and drinking outcomes. Future studies should examine these associations with a longitudinal design to better assess the causality of these variables. Second, the sample was very homogenous in race with the majority identifying as white graduate students. This limits the study’s ability to generalize findings to graduate students from other diverse racial backgrounds. More research is needed to better understand these associations in graduate students from diverse backgrounds. Third, the data for the current study was collected during the fall of 2021, a period that was highly characterized with high levels of COVID-19 and potential impacts on drinking behaviors and patterns. Drinking patterns prior to COVID-19 may be different from what this study found. To increase the validity of these findings, more research should examine graduate student drinking patterns and motives with more recent cohorts. Fourth, the sample showed heterogeneity in ages, field of study, and program types, which may further impact the results of the current study. Research should focus on better understanding potential age differences, in addition to degree type and field type differences among drinking behaviors and motives. All of which could help us better understand the profiles found in the current study and better understand graduate student drinkers. Fifth, our sample size was relatively small compared, limiting our ability to explore a six profile solution. Future studies should aim to recruit a larger sample size to better explore a six profile solution. Sixth, individuals were compared after hard coding their assignment to the most probable latent class rather than conducting regression analyses within the latent profile model, which may affect the accuracy of statistical comparisons between classes (Bucholz et al., 2000). Lastly, the study relied on the use of self-report data, which can influence participants' bias in reporting their drinking behaviors. Although, studies show self-report measures of alcohol use are reliable (Del Boca & Darkes, 2003), future studies should incorporate multimethod modeling approaches such as ecological momentary assessment (EMA) or timeline follow back (TLFB) measures to reduce potential bias (Martin-Willett et al., 2020; Shiffman, 2009).

Findings from the current study can be used to further inform prevention and intervention efforts for graduate students who engage in heavy drinking patterns. Of note, being able to identify the associations between drinking motives and risky drinking patterns, can facilitate the tailoring of interventions to those at the highest risk of problematic use. Interventions should aim to target graduate students who engage in alcohol consumption for conformity or social reasons to help reduce drinks per week and alcohol-related consequences. To further inform intervention strategies, future studies should examine these findings by gender to increase efficacy. In addition, using normative feedback on the association between drinking motives and drinking patterns may aid in increasing the efficacy of personalized feedback interventions^24^.

In conclusion, five distinct profiles of graduate student drinkers were found, based on endorsement of drinking motives, including: those who endorsed 1) moderate levels of all motives, 2) low levels of all motives, 3) high levels of all motives, 4) a mixed level of motives, and 5) high levels of external motives. In addition, External Endorsers reported the highest drinks per week, and High Endorsers reported the highest number of alcohol-related consequences. Low Endorsers reported the lowest drinks per week, and lowest alcohol-related consequences. Our findings suggest that drinking motives are associated with various levels of drinking patterns among graduate students. These findings can be used to target prevention and intervention strategies for students with underlying drinking motives. Interventions tailored to motive-specific risks (e.g., personalized normative feedback, social skills training, or resilience-building programs) should be explored in future studies. In addition, future studies should aim to extend this research examining how these motive based profiles may differ among various variables including gender, race, class year, and academic field.

**References**

Allen, H. K., Barrall, A. L., Beck, K. H., Vincent, K. B., & Arria, A. M. (2020). Situational context and motives of alcohol use among graduate student drinkers. *Addictive behaviors, 104,* 106267.

Allen, H. K., Barrall, A. L., Beck, K. H., Vincent, K. B., & Arria, A. M. (2020). Situational

context and motives of alcohol use among graduate student drinkers. *Addictive Behaviors*, *104*, 106267. <https://doi.org/10.1016/j.addbeh.2019.106267>

American College Health Association. (2019). *American College Health Association-*

*National College Health Assessment II: Reference Group Executive Summary Spring 2019.*

Arnett, J. J. (2000). Emerging adulthood: A theory of development from the late teens through

the twenties. *American psychologist*, *55*(5), 469.

Arnett, J. J. (2007). Emerging adulthood: What is it, and what is it good for?. *Child development*

*perspectives*, *1*(2), 68-73.

Arnett, J. J., & Mitra, D. (2024). COVID-19 and Americans’ Mental Health: A Persistent Crisis,

Especially for Emerging Adults 18 to 29. *Journal of Adult Development,* 1-13. https://doi.org/10.1007/s10804-024-09502-w

Bucholz, K. K., Hesselbrock, V. M., Heath, A. C., Kramer, J. R., & Schuckit, M. A. (2000). A

latent class analysis of antisocial personality disorder symptom data from a multi‐centre family study of alcoholism. Addiction, 95(4), 553-567.

Cadigan, J. M., Martens, M. P., & Herman, K. C. (2015). A latent profile analysis of drinking

motives among heavy drinking college students. *Addictive Behaviors, 51,* 100-105.

Carey, K. B., Henson, J. M., Carey, M. P., & Maisto, S. A. (2010). Perceived Norms Mediate

Effects of a Brief Motivational Intervention for Sanctioned College Drinkers. *Clinical Psychology : A Publication of the Division of Clinical Psychology of the American Psychological Association*, *17*(1), 58–71. https://doi.org/10.1111/j.1468-2850.2009.01194.x

Coffman, D. L., Patrick, M. E., Palen, L. A., Rhoades, B. L., & Ventura, A. K. (2007). Why

Do High School Seniors Drink? Implications for a Targeted Approach to Intervention. *Prevention Science*, *8*(4), 241–248. https://doi.org/10.1007/s11121-007-0078-1

Collins, L. M., & Lanza, S. T. (2009). *Latent class and latent transition analysis: With*

*applications in the social, behavioral, and health sciences* (Vol. 718). John Wiley & Sons.

Collins, R. L., Parks, G. A., & Marlatt, G. A. (1985). Social determinants of alcohol

consumption: the effects of social interaction and model status on the self-administration of alcohol. *Journal of consulting and clinical psychology*, *53*(2), 189.

Cooper, M. (1994). Motivations for Alcohol Use Among Adolescents: Development and

Validation of a Four-Factor Model. *Psychological Assessment*, *6*, 117–128. https://doi.org/10.1037/1040-3590.6.2.117

Cooper, M. (2015). Motivational Models of Substance Use: A Review of Theory and

Research on Motives for Using Alcohol, Marijuana, and Tobacco. In *Oxford Handb. Online*. https://doi.org/10.1093/oxfordhb/9780199381678.013.017

Cooper, M., Krull, J., Agocha, V., Flanagan, M., Orcutt, H., Grabe, S., Dermen, K., & Jackson, M. (2008). Motivational Pathways to Alcohol Use and Abuse Among Black and White Adolescents. *Journal of Abnormal Psychology*, *117*, 485–501. https://doi.org/10.1037/a0012592

Cox, W. M., & Klinger, E. (1988). A motivational model of alcohol use. *Journal of*

*abnormal psychology, 97*(2), 168.

D'Aquino, S., Callinan, S., Smit, K., Mojica-Perez, Y., & Kuntsche, E. (2023). Why do adults

drink alcohol? Development and validation of a Drinking Motives Questionnaire for adults. *Psychology of addictive behaviors, 37*(3), 402. <https://psycnet.apa.org/doi/10.1037/adb0000877>

Del Boca, F. K., & Darkes, J. (2003). The validity of self‐reports of alcohol consumption: state of the science and challenges for research. *Addiction*, *98*, 1-12. https://doi.org/10.1046/j.1359-6357.2003.00586.x

El-Ghoroury, N. H., Galper, D. I., Sawaqdeh, A., & Bufka, L. F. (2012). Stress, coping, and

barriers to wellness among psychology graduate students. *Training and Education in Professional Psychology*, *6*, 122–134. https://doi.org/10.1037/a0028768

Grant, V., Stewart, S., & Mohr, C. (2009). Coping-Anxiety and Coping-Depression Motives

Predict Different Daily Mood-Drinking Relationships. *Psychology of Addictive Behaviors : Journal of the Society of Psychologists in Addictive Behaviors*, *23*, 226–237. https://doi.org/10.1037/a0015006

Holt, L. J., Armeli, S., Tennen, H., Austad, C. S., Raskin, S. A., Fallahi, C. R., Wood, R.,

Rosen, R. I., Ginley, M. K., & Pearlson, G. D. (2013). A person-centered approach to understanding negative reinforcement drinking among first year college students. *Addictive Behaviors*, *38*(12), 2937–2944. https://doi.org/10.1016/j.addbeh.2013.08.015

Ickes, M. J., Brown, J., Reeves, B., & Zephyr, P. M. D. (2015). Differences between

Undergraduate and Graduate Students in Stress and Coping Strategies. *Californian Journal of Health Promotion*, *13*(1), Article 1. https://doi.org/10.32398/cjhp.v13i1.1810

Jochman, K. A., & Fromme, K. (2010). Maturing out of substance use: The other side of

etiology. In *Handbook of drug use etiology: Theory, methods, and empirical findings* (pp. 565–578). American Psychological Association.

Lambert, N. M., Stillman, T. F., Hicks, J. A., Kamble, S., Baumeister, R. F., & Fincham, F. D.

(2013). To Belong Is to Matter: Sense of Belonging Enhances Meaning in Life. *Personality and Social Psychology Bulletin*, *39*(11), 1418–1427. https://doi.org/10.1177/0146167213499186

Lanza, S. T., Bray, B. C., & Collins, L. M. (2012). An introduction to latent class and latent

transition analysis. *Handbook of Psychology, Second Edition,* 2.

Littlefield, A. K., Sher, K. J., & Wood, P. K. (2010). Do changes in drinking motives mediate

the relation between personality change and “maturing out” of problem drinking? *Journal of Abnormal Psychology*, *119*(1), 93–105. https://doi.org/10.1037/a0017512

Martin‐Willett, R., Helmuth, T., Abraha, M., Bryan, A. D., Hitchcock, L., Lee, K., & Bidwell, L.

C. (2020). Validation of a multisubstance online Timeline Followback assessment. *Brain and behavior*, *10*(1), e01486. https://doi.org/10.1002/brb3.1486

Mezquita, L., Stewart, S. H., Ibáñez, M. I., Ruipérez, M. A., Villa, H., Moya, J., & Ortet, G.

(2011). Drinking motives in clinical and general populations. *European addiction research, 17(*5), 250-261.

Offstein, E. H., Larson, M. B., McNeill, A. L., & Mjoni Mwale, H. (2004). Are we doing

enough for today’s graduate student? *International Journal of Educational Management*, *18*(7), 396–407. https://doi.org/10.1108/09513540410563103

Read, J. P., Kahler, C. W., Strong, D. R., & Colder, C. R. (2006). Development and preliminary

validation of the young adult alcohol consequences questionnaire. *Journal of Studies on Alcohol*, *67*(1), 169–177. <https://doi.org/10.15288/jsa.2006.67.169>

Russell, A. M., Vest, N. A., & Litt, D. M. (2022). Social networking site use and alcohol use

behaviors among adolescents: A latent profile analysis. *Addictive behaviors, 129,* 107261. https://doi.org/10.1016/j.addbeh.2022.107261

SAMHSA, (n.d.). *Results from the 2023 National Survey on Drug Use and*

*Health: Detailed Tables, Table 2.9B*. Section 2 PE tables - results from the 2023 National Survey on Drug Use and Health: Detailed tables, Samhsa, CBHSQ. https://www.samhsa.gov/data/sites/default/files/reports/rpt47100/NSDUHDetailedTabs2023/NSDUHDetailedTabs2023/2023-nsduh-detailed-tables-sect2pe.htm#tab2.9b

Shiffman, S. (2009). Ecological momentary assessment (EMA) in studies of substance use.

*Psychological assessment*, *21*(4), 486. https://psycnet.apa.org/doi/10.1037/a0017074

Table 1

*Correlational table with motives, drinks, and consequences*

| No. of profiles | 1 | 2 | 3 | | | 4 | 5 | | 6 | | Mean | SD | | | Min | Max | | |
| --- | --- | --- | --- | --- | --- | --- | --- | --- | --- | --- | --- | --- | --- | --- | --- | --- | --- | --- |
| 1. Social | - | 0.58^*^ | | 0.59^*^ | 0.43^*^ | | 0.29^*^ | 0.17^*^ | | 2.80 | | | 0.74 | 1 | | | 5 |  |
| 2. Cope |  | - | | 0.69^*^ | 0.55^*^ | | 0.48^*^ | 0.25^*^ | | 2.55 | | | 0.75 | 1 | | | 5 |  |
| 3. Enhance |  |  | | - | 0.60^*^ | | 0.52^*^ | 0.21^*^ | | 2.63 | | | 0.68 | 1 | | | 5 |  |
| 4. Conform |  |  | |  | - | | 0.52^*^ | 0.32^*^ | | 2.71 | | | 0.77 | 1 | | | 5 |  |
| 5. Consequences |  |  | |  |  | | **-** | 0.42^*^ | | 7.63 | | | 5.77 | 0 | | | 24 |  |
| 6. Drinks |  |  | |  |  | |  | **-** | | 8.66 | | | 9.03 | 1 | | | 63 |  |

Table 2

*Model fit statistics for 2 to 7 latent profiles*

| No. of profiles | AIC | BIC | Entropy | LMR |
| --- | --- | --- | --- | --- |
| 2 | 3434.54 | 3483.97 | .94 | <0.001 |
| 3 | 3284.71 | 3353.15 | .83 | <0.001 |
| 4 | 3194.25 | 3281.70 | .83 | <0.001 |
| **5** | **3170.91** | **3277.36** | **.86** | **<0.001** |
| 6 | 3111.47 | 3236.94 | .89 | <0.001 |
| 7 | 3120.91 | 3265.39 | .85 | .92 |

*Note:* Bolded row indicated the chosen profile solution; AIC = Akaike’s Information Criterion; BIC = Bayesian Information Criterion; LMR = Lo-Mendell-Rubin

Figure 1

*Scores on each drinking motive dependent on membership in the latent profile for the 5-profile solution.*


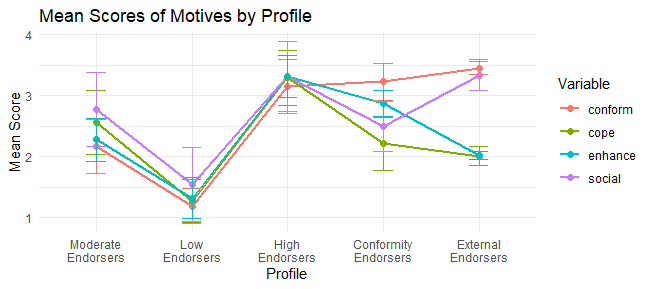


Table 3

*Descriptive statistics of motives and drinking outcomes dependent upon membership in the 5-profile solution*

| Profile | *N* | Social | | | Cope | | | Enhance | | | Conform | | |
| --- | --- | --- | --- | --- | --- | --- | --- | --- | --- | --- | --- | --- | --- |
|  |  | *M* | *SD* | *M* | | *SD* | *M* | | *SD* | *M* | | *SD* |  |
| 1 | 102 | 2.77 | .60 | 2.56 | | .51 | 2.29 | | .36 | 2.18 | | .46 |  |
| 2 | 29 | 1.56 | .61 | 1.27 | | .36 | 1.33 | | .35 | 1.18 | | .26 |  |
| 3 | 100 | 3.35 | .57 | 3.31 | | .44 | 3.32 | | .35 | 3.18 | | .45 |  |
| 4 | 80 | 2.39 | .49 | 2.37 | | .37 | 2.77 | | .23 | 3.14 | | .29 |  |
| 5 | 20 | 3.39 | .11 | 2.02 | | .15 | 2.02 | | .06 | 3.44 | | .11 |  |

*Note:* Profile 1 = moderate endorsers; Profile 2 = low endorsers; Profile 3 = high endorsers; Profile 4 = conformity endorsers; Profile 5 = external endorsers

Table 4

*Tukey’s Post-Hoc Analyses For Drinking Outcomes According to Profile Membership*

| Outcome variable | Comparison | Mean Difference | SE | p-value |
| --- | --- | --- | --- | --- |
| Drinks per week | 1-2 | 4.23 | .46 | < .001 |
|  | 1-3 | -3.37 | .08 | < .01 |
|  | 1-5 | -2.73 | .11 | < .05 |
|  | 2-3 | -6.28 | .06 | < .001 |
|  | 2-4 | -4.92 | .07 | < .001 |
|  | 2-5 | -5.42 | .06 | < .001 |
| Alcohol-related consequences | 1-2 | 6.40 | 1.03 | < .001 |
|  | 1-3 | -6.46 | .06 | < .001 |
|  | 2-3 | -9.37 | .02 | < .001 |
|  | 2-4 | -9.37 | .04 | < .001 |
|  | 2-5 | -8.33 | .05 | < .001 |
|  | 3-4 | 1.70 | .35 | < .01 |
|  | 3-5 | 3.54 | .24 | .05 |

*Note:* Profile 1 = Moderate Endorsers; Profile 2 = Low Endorsers; Profile 3 = High Endorsers; Profile 4 = Conformity Endorsers; Profile 5 = External Endorsers
